# Diseases of the Digestive Organs of Infancy and Childhood

**Published:** 1891-09

**Authors:** 


### Book Reviews.

Diseases of the Digestive Organs of Infancy and Childhood. By Louis Starr, M. D. Blakiston, Son & Co. Philadelphia

This popular volume has passed into its second edition with a decided increase in the instructive matter. The author has endeavored to bring the subject matter fully abreast with the times. In doing so, he has added some very important subjects, such as odor of the breath in disease, massage, etc.; also, deals more fully than many larger works of the kind, on the troubles associated with and due to the process of dentition. The book is neatly gotten up, and should be carefully read by all who are interested in the diseases of childoood.

